# Use of carbapenems and glycopeptides increases risk for *Clostridioides difficile* infections in acute myeloid leukemia patients undergoing intensive induction chemotherapy

**DOI:** 10.1007/s00277-020-04274-1

**Published:** 2020-09-24

**Authors:** Olivier Ballo, Eva-Maria Kreisel, Fagr Eladly, Uta Brunnberg, Jan Stratmann, Peter Hunyady, Michael Hogardt, Thomas A. Wichelhaus, Volkhard A. J. Kempf, Björn Steffen, Joerg J. Vehreschild, Maria J. G. T. Vehreschild, Fabian Finkelmeier, Hubert Serve, Christian H. Brandts

**Affiliations:** 1 Department of Medicine, Hematology/Oncology, University Hospital, Goethe University, Theodor-Stern-Kai 7, 60596 Frankfurt/Main, Germany; 2 Department of Medicine, Gastroenterology, Hepatology and Endocrinology, University Hospital, Goethe University, Theodor-Stern-Kai 7, Frankfurt/Main, Germany; 3 Institute of Medical Microbiology and Infection Control, Goethe University Hospital, Theodor-Stern-Kai 7, Frankfurt/Main, Germany; 4 University Center for Infectious Diseases, Goethe University Hospital, Theodor-Stern-Kai 7, Frankfurt/Main, Germany; 5 University Center of Competence for Infection Control, Frankfurt - Giessen -, Marburg, Hessen Germany; 6 German Cancer Consortium (DKTK) and German Cancer Research Center (DKFZ), Heidelberg, Germany; 7 Department of Internal Medicine, Infectious Diseases, University Hospital Frankfurt, Goethe University, Theodor-Stern-Kai 7, Frankfurt/Main, Germany; 8 Frankfurt Cancer Institute, Goethe University Frankfurt, Theodor-Stern-Kai 7, Frankfurt/Main, Germany; 9 University Cancer Center Frankfurt (UCT), University Hospital, Goethe University, Theodor-Stern-Kai 7, Frankfurt/Main, Germany

**Keywords:** AML, CDI, Induction chemotherapy

## Abstract

Patients with acute myeloid leukemia (AML) are often exposed to broad-spectrum antibiotics and thus at high risk of *Clostridioides difficile* infections (CDI). As bacterial infections are a common cause for treatment-related mortality in these patients, we conducted a retrospective study to analyze the incidence of CDI and to evaluate risk factors for CDI in a large uniformly treated AML cohort. A total of 415 AML patients undergoing intensive induction chemotherapy between 2007 and 2019 were included in this retrospective analysis. Patients presenting with diarrhea and positive stool testing for toxin-producing *Clostridioides difficile* were defined to have CDI. CDI was diagnosed in 37 (8.9%) of 415 AML patients with decreasing CDI rates between 2013 and 2019 versus 2007 to 2012. Days with fever, exposition to carbapenems, and glycopeptides were significantly associated with CDI in AML patients. Clinical endpoints such as length of hospital stay, admission to ICU, response rates, and survival were not adversely affected. We identified febrile episodes and exposition to carbapenems and glycopeptides as risk factors for CDI in AML patients undergoing induction chemotherapy, thereby highlighting the importance of interdisciplinary antibiotic stewardship programs guiding treatment strategies in AML patients with infectious complications to carefully balance risks and benefits of anti-infective agents.

## Introduction

Acute myeloid leukemia (AML) is one of the most aggressive hematological malignancies arising from transformed myeloid precursor cells. Curative treatment with induction chemotherapy is due to its high toxicity only initiated in patients with no or only limited comorbidities up to 60–75 years of age [1–3]. Disease- and therapy-related immunosuppressions going along with extensive use of broad-spectrum antibiotics predispose AML patients for nosocomial infectious diarrhea. *Clostridioides difficile* is the most frequently found bacterial pathogen in AML patients suffering from infectious diarrhea [4].

Higher age, use of antibiotics, sepsis, acute renal failure (ARF), duration of neutropenia, length of hospital stay, and AML as the underlying disease have been identified as risk factors for ***Clostridioides difficile*** **infections** (CDI) in leukemia patients [4–6]. Furthermore, CDI has been shown to be a relevant infectious complication in AML patients undergoing allogenic stem cell transplantation (SCT), increasing gastro-intestinal graft-versus-host disease, and non-relapse mortality [7, 8]. We conducted this retrospective study in a large homogenously treated AML patient cohort (a) to analyze the incidence of CDI at the University Hospital Frankfurt and its impact on the clinical course of induction chemotherapy and (b) to identify factors associated with CDI.

## Materials and methods

### Study design and treatment protocols

In this single-center study, we retrospectively included all patients with AML who underwent intensive induction chemotherapy between 2007 and 2019. Screening period for CDI included only the hospital stay for induction chemotherapy. Standard induction chemotherapy was the so-called 7 + 3-regime, cytarabine 100 mg/m^2^ given for 7 days combined with daunorubicin 60 mg/m^2^ given for 3 days [9]. In general patients under the age of 60 received a second induction therapy with 7 + 3, if early blast clearance was achieved on d15 bone marrow blood evaluation or with a salvage protocol “HAM” (cytarabine 3000 mg/m^2^ every 12 h for 3 days and mitoxantrone 10 mg/m^2^ for 3 days) and if blast clearance was not achieved on d15 bone marrow blood evaluation [10]. Patients above the age of 60 received only a second induction chemotherapy with HAM (with reduced cytarabine dose of 1000 mg/m^2^), if the first induction therapy cycle was not sufficient to achieve bone marrow blast clearance on d15 [11]. All patients received routinely antimicrobial prophylaxis with levofloxacin and posaconazole as suggested by current guidelines [12, 13]. If fever or a significant increase of C-reactive protein (CRP) was found, antibiotic prophylaxis was replaced by intravenous broad-spectrum antibiotics.

The study was performed in accordance with the 2013 Declaration of Helsinki [14]. Patients provided written informed consent to retrospective data extraction from patient charts, and patient data was provided after approval by the local Ethics Committee (approval number SHN-08-2019). After ethics approval, data from all AML patients receiving intensive induction chemotherapy at the University Hospital Frankfurt was retrieved from the clinical cancer registry of the University Cancer Center (UCT) Frankfurt and complemented by data directly from the patients archived medical records. Data analysis was performed on anonymized data.

### Definitions of CDI

A patient presenting with diarrhea (≥ 3 loose stools within 24 h) and a stool sample positive for *C. difficile* toxin or positive for toxin-producing *C. difficile* was defined to have CDI [15]. From 2007 until 2017 microbiological laboratory diagnosis of CDI was based on positive *C. difficile* toxin assay (C. difficile TOX A/B II™, Alere, Germany) and/or the cultural detection of toxigenic *C. difficile.* For toxinogenic *C. difficile* culture, stool specimens were inoculated onto cycloserine-cefoxitin-fructose agar (CCFA; Oxoid, Wesel, Germany) and incubated at 37 °C for 48 h. Identification of *C. difficile* was performed by matrix-assisted-laser desorption ionization-time of flight mass spectrometry (VITEK MS, bioMérieux, Nürtingen, Germany). Culture isolates of *C. difficile* were tested for toxin producing by using TOX A/B II EIA from culture supernatants (toxinogenic culture). Since 2017, fecal samples were investigated for *C. difficile*–specific glutamate dehydrogenase (GDH) by an enzyme immunoassay (*C. Diff* Chek-60™, Alere, Germany) according to updated guidelines for CDI diagnosis [16]. Samples with a negative test result were reported negative; positive samples were tested for the presence of free *C. difficile* toxins A and B or the toxin B gene *tcdB* (BD MAX™ Cdiff assay, Becton Dickinson, Heidelberg). If only GDH and *C. difficile* TcdB gene are present, CDI cannot be differentiated from asymptomatic colonization (the latter did not occur in the study population).

### Statistical analysis

Continuous variables are shown as means ± standard deviation, and categorical variables are reported as frequencies and percentages. All continuous variables were tested for normality and were analyzed by using the Student’s *t* test or the Wilcoxon–Mann–Whitney test accordingly. Chi-squared test was used for binary variables. Risk factors for CDI were determined using a univariate and multivariate binary logistic regression model. For assessment of survival factors, we used a univariate and multivariate cox-regression model. All *p* values reported are two-sided. Statistical significance was assumed when the *p* value was < 0.05. Statistical analysis was performed with SPSS (Version 22.0, IBM, Armonk, NY).

## Results

### Baseline characteristics

Of 415 AML patients 37 (8.9%) suffered from CDI during the hospital stay of induction chemotherapy and 378 AML patients (91.1%) had no evidence of CDI during that time. Median age was 58 years (range 22–76) in AML patients with CDI and 59 years (range 18–85) in AML patients without CDI (*p* = 0.701). There was no significant difference between both cohorts with respect to sex, AML subtypes, and AML risk groups (Table 1) [17, 18].

**Table 1:** Baseline characteristics

Characteristic	All	AML with CDI	AML without CDI	*p* value
Number of patients (*n*, %)	415 (100)	37 (8.9)	378 (91.1)	
Median age (median, range)	59 (18-85)	58 (22-76)	59 (18-85)	0.701
Female sex (*n*, %)	190 (45.8)	17 (45.9)	137 (36.2)	0.562
AML with recurrent genetic abnormalities (*n*, %)	176 (42.4)	18 (48.6)	158 (41.8)	0.925
AML with myelodysplasia-related changes (*n*, %)	56 (13.5)	4 (10.8)	52 (13.8)	0.925
Therapy-related myeloid neoplasms (*n*, %)	5 (1.2)	0 (0)	5 (1.3)	0.925
AML not otherwise specified (*n*, %)	175 (42.2)	15 (40.5)	160 (42.3)	0.925
**Acute leukemias of ambiguous lineage** (*n*, %)	1 (0.2)	0 (0)	1 (0.2)	0.925
Myeloid sarcoma (*n*, %)	1 (0.2)	0 (0)	1 (0.2)	0.925
Favorable ELN risk group (*n*, %)	85 (20.5)	7 (18.9)	78 (20.6)	0.782
Intermediate-I ELN risk group (*n*, %)	157 (37.8)	12 (32.4)	145 (38.4)	0.782
Intermediate-II ELN risk group (*n*, %)	91 (21.9)	10 (27.0)	81 (21.4)	0.782
Adverse ELN risk group (*n*, %)	75 (18.1)	8 (21.6)	67 (17.7)	0.782

All *p* values reported are two-sided. Statistical significance was defined as *p* ≤ 0.05

### Clinical findings and outcome in AML patients with and without CDI

A total of 25 (67.6%) of the 37 AML patients with CDI were diagnosed between 2007 and 2012 and 12 (32.4%) between 2013 and 2019, whereas 169 (43.1%) of 386 AML patients without CDI were diagnosed between 2007 and 2012 and 207 (56.8%) between 2013 and 2019 (*p* = 0.009). Thus, the CDI rate was 13.3% for AML patients diagnosed in 2007–2012 and 5.3% for AML patients diagnosed in 2013–2019. There was no difference between AML patients with and without CDI with respect to the length of the hospital stay for induction chemotherapy (49 days vs. 49 days, *p* = 0.454). AML patients with CDI had a median of 7 (0–28) days with fever compared with 5 (0–31) days with fever in AML patients without CDI (*p* = 0.048); median CRP levels were 4.58 (0.39–19.42) in AML patients with CDI and 3.99 (0.19–34.66) in AML patients without CDI (*p* = 0.312). Seven (18.9%) AML patients with CDI and 67 (17.7%) AML patients without CDI required treatment on ICU (*p* = 0.824); acute renal failure (ARF) was seen in about 19% of both cohorts (*p* = 0.824). Complete remission (CR) and allogenic SCT rates as consolidation therapy were similar in both cohorts. At the time of this analysis 18 (48.6%) AML patients with CDI and 174 (46.0%) AML patients without CDI were still alive (*p* = 0.863) (Table 2).

**Table 2:** Clinical findings in AML patients with and without CDI

Characteristic	AML with CDI	AML without CDI	*p* value
Number of patients (*n*, %)	37 (8.9)	378 (91.1)	
Diagnosed between 2007 and 2012 (*n*, %)	25 (67.6)	163 (43.1)	0.005
Diagnosed between 2013 and 2019 (*n*, %)	12 (32.4)	215 (56.8)	0.005
Length of hospital stay (median, range)	49 (28-82)	49 (5–127)	0.386
Days with fever (median, range)	7 (0-28)	5 (0–31)	0.048
C-reactive protein (median, range)	4,58 (0.39–19.42)	3.99 (0.19–34.66)	0.312
Patients requiring treatment on intensive care unit (*n*, %)	7 (18.9)	67 (17.7)	0.824
Incidence of acute renal failure (*n*, %)	7 (18.9)	71 (18.8)	1.000
Complete remission after induction chemotherapy (*n*, %)	25 (67.6)	236 (62.4)	0.722
Allogenic stem cell transplantation as consolidation therapy (*n*, %)	19 (51.4)	211 (55.8)	0.608
Overall survival (*n*, %)	18 (48.6)	174 (46.0)	0.863

All *p* values reported are two-sided. Statistical significance was defined as *p* ≤ 0.05

### Distribution of anti-infective agents in AML patients with and without CDI

The distribution of anti-infective agents used in AML patients with and without CDI is illustrated in Table 3. AML patients with CDI had a higher median exposure to antibiotics (cumulative calculation for fluoroquinolones, acylaminopenicillins with ß-lactamase inhibitor (BLI), carbapenems, and glycopeptides) than AML patients without CDI (70 vs. 59, *p* = 0.027). In subgroup analyses for the different antibiotics, AML patients with CDI had a significantly longer exposure to carbapenems than those without (28 days, range 0–50 vs. 17 days, range 0–72, *p* = 0.001). AML patients with CDI also had a significantly higher exposure to glycopeptides than those without (18 days, range 0–63 vs. 11 days, range 0–51, *p* ≤ 0.0001). On the other hand, AML patients with CDI had less exposure to acylaminopenicillins with BLI, being 0 days (0–23) in AML patients with CDI and 7 days (0–56) in AML patients without CDI (*p* = 0.009). Exposure to fluoroquinolones and cumulative exposure to antifungals were similar in both cohorts.

**Table 3:** Distribution of anti-infective agents in AML patients with and without CDI

Characteristic	AML with CDI	AML without CDI	*p* value
Number of patients (*n*, %)	37 (8.9)	378 (91.1)	
Cumulative antibiotic exposure to fluoroquinolone, acylaminopenicillin + ß-lactamase inhibitor, carbapenem and glycopeptide (median, range)*	70 (29–125)	59 (2–169)	0.027
Cumulative fluoroquinolone exposure (median, range)*	17 (0–38)	20 (0–78)	0.460
Cumulative acylaminopenicillin + ß-lactamase inhibitor exposure (median, range)*	0 (0–23)	7 (0–56)	0.009
Cumulative carbapenem exposure (median, range)*	28 (0–50)	17 (0–72)	0.001
Cumulative glycopeptide exposure (median, range)*	18 (0–63)	11 (0–51)	< 0.0001
Cumulative exposure to antifungals (median, range)*	34 (9–54)	32.5 (0–178)	0.319

*Measured in days of therapy, multiple antibiotics given on the same day were counted as multiple antibiotic days. All *p* values reported are two-sided. Statistical significance was defined as *p* ≤ 0.05

To further analyze days with fever and the exposition to different anti-infective agents as risk factors for CDI in AML patients undergoing induction chemotherapy, a binary logistic regression model with forward stepwise likelihood ratio was performed. The nominal dichotome variables, female sex, age > 60, days with fever, cumulative antibiotic exposure to fluoroquinolones, acylaminopenicillin with BLI, carbapenems and glycopeptides, cumulative fluoroquinolone exposure, cumulative acylaminopenicillin with BLI exposure, cumulative carbapenem exposure, and cumulative glycopeptide exposure, were included in this model. As shown in Table 4 in a multivariate analysis exposure to glycopeptides was found to be an independent risk factor for CDI in AML patients undergoing induction chemotherapy (odds ratio (OR) = 1.055, 95% confidence interval (CI) 1.010–1.102, *p* = 0.016).

**Table 4:** Univariate and multivariate analysis (all included with *p* < 0.1) associated with CDI in AML patients

Parameter	OR	95% CI	*P* value	OR	95 % CI	*p* value
	Univariate analysis			Multivariate analysis		
Female sex	1.007	0.512–1.983	0.983			
Age > 60	1.232	0.623–2.434	0.549			
Days with fever	1.048	0.999–1.101	0.057			
Cumulative antibiotic exposure to fluoroquinolones, acylaminopenicillin + ß-lactamase inhibitors, carbapenems and glycopeptides*	1.012	1.000–1.024	0.059			
Cumulative fluoroquinolone exposure*	0.959	0.959–1.014	0.986			
Cumulative acylaminopenicillin + ß-lactamase inhibitor exposure (median, range)*	0.953	0.911–0.996	0.034			
Cumulative carbapenem exposure*	1.029	1.009–10.51	0.005			
Cumulative glycopeptide exposure*	1.053	1.023–1.083	< 0.001	1.055	1.010–1.102	0.016

*Measured in days of therapy, multiple antibiotics given on the same day were counted as multiple antibiotic days. CI indicates confidence interval and HR hazard ratio. All *p* values reported are two-sided. Statistical significance was defined as *p* ≤ 0.05

### Treatment results for CDI

A total of 34 AML patients (91.9%) received treatment for CDI of which 23 (62.2%) were treated with metronidazole orally/intravenously only, 3 (8.1%) with vancomycin orally only (Table 5). A total of 7 (18.9%) AML patients with CDI were initially treated with metronidazole; treatment was then replaced or extended with vancomycin due to intolerance or inefficacy of metronidazole. The median time to treatment response was 7 (range 3–12) for metronidazole, 11 (range 6–19) for vancomycin, and 12 (range 8–27) days for metronidazole- and/or vancomycin-treated AML patients, respectively. Treatment response on day 10 was achieved for 8 (34.8%) metronidazole-treated AML patients, for 1 (33.3%) vancomycin-treated AML patient, and for 1 (14.3%) AML patient treated with both, while recurrent CDI within 90 days was seen in 8 (34.8%), 0 (0%), and 2 (28.6%) AML patients, respectively (cumulative recurrence rate of 27%). One patient received treatment with fidaxomicin, responded to treatment on day 6, and had no recurrent CDI within 90 days. In 11 AML patients with CDI a diagnostic computed tomography scan of the abdomen was performed; in 5 AML patients bowel wall thickening and ascites were seen.

**Table 5:** Efficacy of CDI treatment in AML patients

Characteristic	All	Treatment with metronidazole only	Treatment with vancomycin only	Treatment with metronidazol and/or vancomycin	Treatment with fidaxomicin	No treatment
Number of patients (*n*, %)	37 (100)	23 (62.2)	3 (8.1)	7 (18.9)	1 (2.7)	3 (8.1)
Days to treatment response (median, range)	7 (3–12)	7 (3–12)	11 (6–19)	12 (8–27)	6	2 (0–3)
Treatment response day 10 (*n*, %)	11 (29.7)	8 (34.8)	1 (33.3)	1 (14.3)	1 (100)	3 (100)
Recurrent CDI within 90 days (*n*, %)	10 (27.0)	8 (34.8)	0 (0)	2 (28.6)	0 (0)	0 (0)

All *p* values reported are two-sided. Statistical significance was defined as *p* ≤ 0.05

## Discussion

In this study, we analyzed the incidence of CDI in a large uniformly treated AML cohort undergoing intensive induction chemotherapy at the University Hospital Frankfurt. Overall, 37 (8.9%) of 415 included AML patients suffered from CDI during the hospital stay for induction chemotherapy. The CDI rate of 8.9% in our AML cohort is in accordance with a CDI rate of 8.62% reported by Vehreschild et al. at the University Hospital of Cologne for AML patients at first hospitalization when censored for patients receiving curative chemotherapy [19]. Schalk et al. reported a higher CDI rate per AML patient at the University Hospital Magdeburg (18%). However, here AML patients were analyzed for CDI throughout several hospitalizations and repeated chemotherapy courses [4]. Ford et al. analyzed CDI in 509 consecutive patients with newly diagnosed acute leukemia at the LDS Hospital in Salt Lake City and found only 31 leukemia patients (6%) to have CDI [20]. However, 7% of these 509 leukemia patients did not receive induction chemotherapy treatment and 21% of these patients did not have AML as their underlying disease (being itself a risk factor for CDI amongst patients with acute leukemia). Considering the individual study populations, the CDI rate in our hematology department is in accordance with the CDI rates described by other studies.

To identify a possible trend towards increasing or decreasing CDI rates in our hematology department, we analyzed CDI rates by splitting our AML cohort into two subgroups, one including AML patients diagnosed between 2007 and 2012 and the other one including AML patients diagnosed between 2013 and 2019. At our institute the CDI rate in AML patients decreased from 13.3% during 2007–2012 to 5.3% during 2013–2019. This observation is in accordance with a recently published meta-analysis by Ho et al. revealing decreasing CDI rates in most European countries between 2005 and 2015 [21].

In our analysis, CDI did not adversely affect the clinical course of AML patients undergoing induction chemotherapy. Although AML patients with CDI had in median 2 more days with fever than AML patients without CDI (7 vs. 5 days, *p* = 0.048), relevant clinical factors such as length of the hospital stay or need for treatment on ICU were not significantly influenced and median CRP levels were similar in both cohorts. Acute renal failure (ARF) (known as an independent marker of CDI severity) was nearly 19% in both cohorts [22, 23]. Still, ARF is a frequent complication of CDI and demands attention in treatment of AML patients with CDI and with diarrhea in general.

Anti-infective agents used in therapy-refractory infections such as carbapenems and glycopeptides were more frequently used in AML patients with CDI. The finding of carbapenem use as a risk factor for CDI in AML patients is in accordance with the study by Vehreschild et al. [19]. An association between glycopeptides and CDI in AML patients has also been observed by others [7, 24]. This is the first study to analyze the use of anti-fungal medication in the context of CDI in AML patients. We found exposition to antifungal medications not to be significantly associated with CDI.

Since the late 1990s metronidazole has been recommended as the first choice for treatment of uncomplicated CDI [25]. For patients with severe CDI, a randomized controlled trial has shown superiority of vancomycin over metronidazole [26]. The most frequently chosen treatment for CDI in our study was metronidazole used in 23 patients (62.2%). Only 3 AML patients (8.1%) with CDI received vancomycin as 1st line treatment. Response rate on day 10 was 34.8% (*n* = 3) in the metronidazole-treated CDI and 33.3% (*n* = 1) in the vancomycin treated CDI, but recurrent CDI within 90 days was seen in 34.8% (*n* = 8) and 0% (*n* = 0), respectively. One patient was treated with fidaxomicin and had no CDI recurrence. Due to the small number of patients treated for CDI no meaningful conclusions can be drawn from this data.

In summary, we found a CDI rate at our hematology department consistent with the incidence reported by other studies. Carbapenems and glycopeptides that are highly important antimicrobial agents especially for patients with sepsis or therapy-refractory infections have been confirmed to be a risk factor for CDI in AML patients in this study, whereas no association between CDI and ARF, treatment on ICU, antifungal medication, or survival was observed. This study highlights the importance of interdisciplinary antibiotic stewardship programs for guiding treatment strategies in AML patients with challenging therapy-refractory infectious complications to carefully balance the risks and benefits of intensive anti-infective agents.
